# Intrapopulation differences in polar bear movement and step selection patterns

**DOI:** 10.1186/s40462-022-00326-5

**Published:** 2022-05-23

**Authors:** Ryan R. Wilson, Michelle St. Martin, Eric V. Regehr, Karyn D. Rode

**Affiliations:** 1 U.S. Fish and Wildlife Service, Marine Mammals Management, Anchorage, AK USA; 2 Polar Science Center, University of Washington, Seattle, WA USA; 3 U.S. Geological Survey, Alaska Science Center, Anchorage, AK USA; 4 Present Address: U.S. Fish and Wildlife Service, Portland, OR 97266 USA

**Keywords:** Age class, Chukchi Sea subpopulation, Movement, Polar bear, Spatial ecology, Sex, Step selection, *Ursus maritimus*

## Abstract

**Background:**

The spatial ecology of individuals often varies within a population or species. Identifying how individuals in different classes interact with their environment can lead to a better understanding of population responses to human activities and environmental change and improve population estimates. Most inferences about polar bear (*Ursus maritimus*) spatial ecology are based on data from adult females due to morphological constraints on applying satellite radio collars to other classes of bears. Recent studies, however, have provided limited movement data for adult males and sub-adults of both sexes using ear-mounted and glue-on tags. We evaluated class-specific movements and step selection patterns for polar bears in the Chukchi Sea subpopulation during spring.

**Methods:**

We developed hierarchical Bayesian models to evaluate polar bear movement (i.e., step length and directional persistence) and step selection at the scale of 4-day step lengths. We assessed differences in movement and step selection parameters among the three classes of polar bears (i.e., adult males, sub-adults, and adult females without cubs-of-the-year).

**Results:**

Adult males had larger step lengths and less directed movements than adult females. Sub-adult movement parameters did not differ from the other classes but point estimates were most similar to adult females. We did not detect differences among polar bear classes in step selection parameters and parameter estimates were consistent with previous studies.

**Conclusions:**

Our findings support the use of estimated step selection patterns from adult females as a proxy for other classes of polar bears during spring. Conversely, movement analyses indicated that using data from adult females as a proxy for the movements of adult males is likely inappropriate. We recommend that researchers consider whether it is valid to extend inference derived from adult female movements to other classes, based on the questions being asked and the spatial and temporal scope of the data. Because our data were specific to spring, these findings highlight the need to evaluate differences in movement and step selection during other periods of the year, for which data from ear-mounted and glue-on tags are currently lacking.

**Supplementary Information:**

The online version contains supplementary material available at 10.1186/s40462-022-00326-5.

## Background

The spatial ecology of individuals can vary within a species [1] with different classes often exhibiting distinct space use and movement patterns [2–4]. Multiple factors can lead to individual variation in a population, such as physical and behavioral development [5], social status [6], and life history stage [7]. These differences are often the result of variation in ecological constraints among classes (e.g., sex, age, reproductive status). For example, an individual’s size or age can have an effect on diet [8] which can lead to different movement decisions for accessing their desired prey or forage [9].

Frequently, location data are obtained for one class of animal in a population and used to make inference to the entire population [10, 11]. Depending on the questions being posed, monitoring the movements of only one class (e.g., adult females) may be justified (e.g., [12]). If, however, the questions are related to the overall ecology of the population (e.g., disease transmission [13]), then a more holistic approach may be required. This is especially true if studies have not previously established that different classes of animals exhibit similar movement decisions and patterns. Assuming that one class is representative of others can lead to erroneous ecological inference or inefficient management and conservation strategies [14].

Polar bears (*Ursus maritimus*) embody many of the challenges listed above. Movement data to inform polar bear management and conservation are nearly always obtained from adult females due to morphological issues with collar retention on adult males and concerns about collars on sub-adults becoming too tight as bears grow [15]. As a result, most inferences about polar bear space use decisions and movement patterns are derived from adult females [16–19]. A few studies have analyzed differences in the spatial ecology among different classes of polar bears [20–22], but the logistical challenges of collecting these data make such analyses rare. Application of these results to other classes of bears typically requires an assumption that all classes of bears use space similarly [18, 23], but the few studies where animals other than adult females have been monitored show this assumption may be incorrect [20–22].

Understanding the spatial ecology of polar bear classes other than adult females can provide valuable insights into their ecology and the efficacy of management actions. Studies have shown that resource selection patterns of adult female polar bears are largely invariant to climate-induced changes to sea ice [18, 24]. It remains unclear, however, if other classes of bears (i.e., adult males, sub-adults) use space differently, and if so, how their responses to sea-ice loss might differ from adult females. Data on movements and space use of adult female polar bears have also been critical to estimating demographic parameters of polar bear subpopulations [23]. If adult female movements and space use are significantly different from other classes of polar bears, then these demographic estimates might be biased in an unknown direction.

Previous research has shown that sub-adult polar bears are typically most responsible for human-polar bear conflicts [25, 26]. Nutritionally-stressed adult males have also been implicated in higher rates of attacks on humans than adult females [26]. Studies on movement and space use decisions of sub-adult and adult male bears can therefore help understand when and where polar bears are most likely to interact with humans. With sea-ice loss increasing and leading to more bears on land for longer periods each year [16, 17], conflicts with polar bears are likely to increase [26]. Thus, information on the space use decisions and movement patterns of adult males and sub-adults, compared to adult females, can provide insight into how conflicts with humans might be mitigated.

Given the difficulty of tracking the movements of polar bears other than adult females, it is desirable to identify if adult female space use decisions and movement patterns differ sufficiently from other classes such that their use as a proxy is unwarranted. Since 2010, adult males and sub-adults have been tagged with satellite telemetry ear-tags or glue-on tags during spring live-capture research in the Chukchi Sea (CS) which provide location data for approximately 3 months allowing an understanding of polar bear movements during a critical feeding period for polar bears [27]. Our objective in this study was to determine whether differences exist in key movement metrics (i.e., directional persistence, step lengths) and step selection patterns between adult females, adult males, and sub-adults. Given the observed differences in diet between adult males and other classes of polar bears [27], and the differences in spatial distribution of prey [28], we predicted that step selection patterns would differ between adult males and adult females and sub-adults. Because movement patterns of adult males are thought to be related to mate-finding behavior in spring [21], we also predicted that adult males would have less directional persistence and similar step lengths as adult females [21] and sub-adults.

## Methods

### Study area

**Fig. 1:**
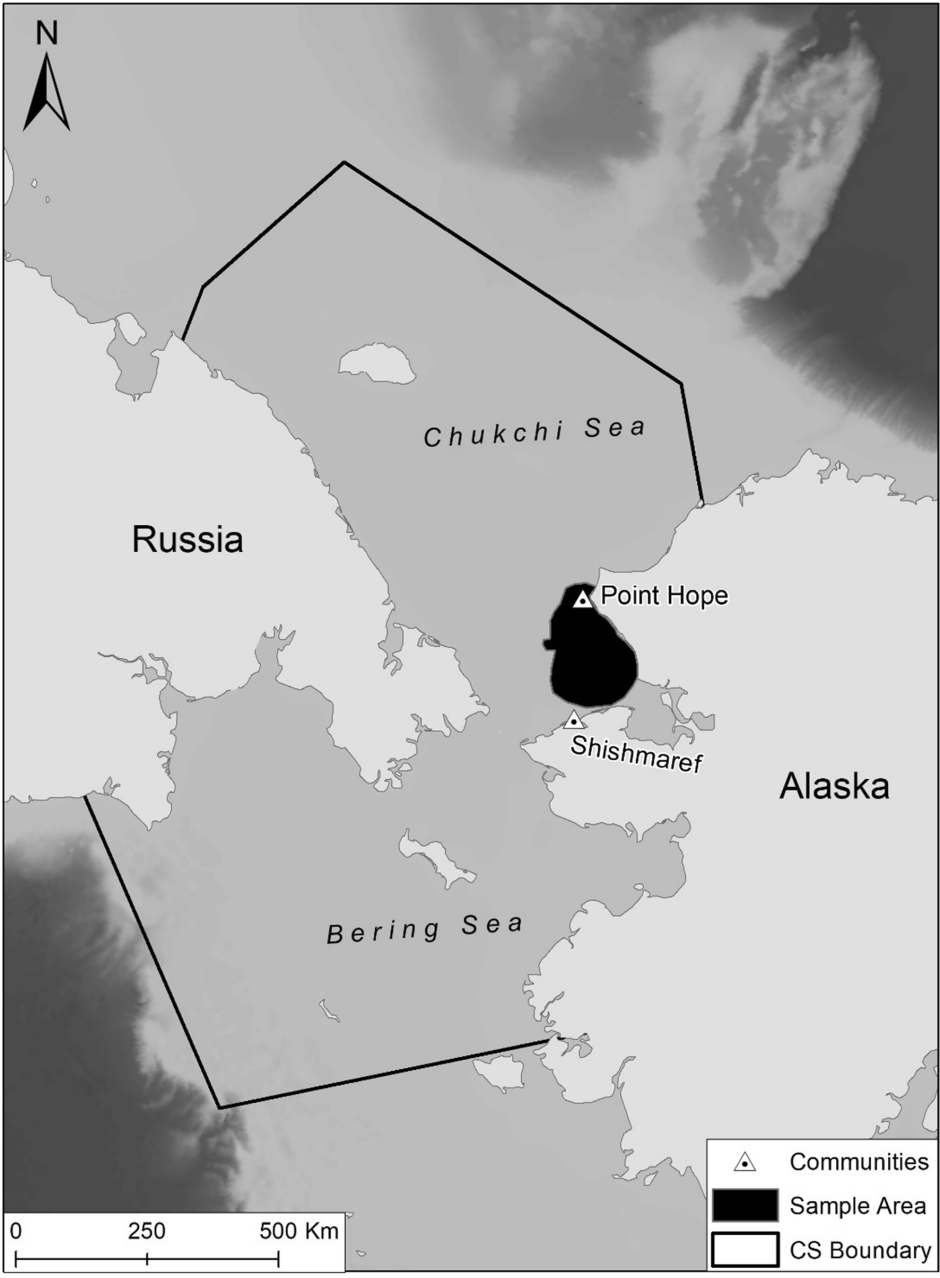
Map depicting the boundary for the Chukchi Sea (CS) subpopulation (black line) as defined by the Polar Bear Specialists Group and the region where polar bear captures occurred for this study (the black “sample area” polygon) between the communities of Point Hope and Shishmaref, Alaska. The gray shading in the ocean depicts the ocean depth across the study area, with darker regions indicating deeper waters

The CS subpopulation is one of 19 recognized polar bear subpopulations [29] ranging from northwestern Alaska to northeastern Chukotka, Russia (Fig. 1). The CS subpopulation’s range [29] is bounded by the Bering Sea to the south, the East Siberian Sea to the west, the Beaufort Sea to the east, and the continental shelf to the north (Fig. 1), representing an area of approximately 1,600,000 km^2^. Sea ice in the CS is highly dynamic with ice forming, shifting, and melting throughout the year. Sea ice in the CS currently reaches its maximum extent in March and its minimum extent in September [30]. Polar bears in the CS subpopulation primarily prey on ringed (*Pusa hispida*) and bearded seals (*Erignathus barbatus*), with spring being a critical hunting period [27].

### Animal capture

We captured polar bears from a helicopter and immobilized them with a dart containing zolazepam-tiletamine (Telazol or Zoletil; [31]) between mid-March and late April (2010–2017, excluding 2012 and 2014) in U.S. waters between Point Hope and Shishmaref, Alaska (Fig. 1). We fitted captured adult females with a GPS collar that was set to drop off after 12–15 months. Our sample of adult females did not include any females with cubs-of-the-year as these bears were not available to be captured in our study area given that most bears in the subpopulation den on Wrangel Island [17], approximately 600 km northwest. Similarly, we did not include data from bears collared the previous year that had denned. As noted earlier, we did not fit GPS collars to male bears or sub-adults. Instead, we fit these bears with an Argos satellite telemetry tag affixed to either an ear or glued onto their back.

### Location data

Non-collar satellite-based transmitters for polar bears typically have poor performance [15], with a maximum life span often lasting < 6 months [15, 20, 21]. The poor performance of these devices are thought to be related to tags being lost (e.g., pulled out of ear or lost due to molting), antennae failure, and limited battery life [15]. Our comparative analyses were therefore limited to location data obtained between 1 March and 30 June, when the three classes of bears had active tags. To ensure that capturing bears did not influence their movements, we restricted each bear’s data to ≥ 5 days post-capture [32]. Even though we fit adult females with GPS collars, preliminary analyses found that models had difficulty converging when using GPS data from adult females and Argos data from other classes, even if GPS data were subset to match the acquisition intervals from the Argos tags. This was likely due to large differences in uncertainty for GPS-based locations compared to Argos-based locations [33]. Because GPS collars also collected Argos locations, we only used Argos locations for adult females. Given that Argos transmitters can provide multiple locations during a duty cycle, we restricted Argos data to only one location per duty cycle (i.e., satellite acquisition period), keeping the location with the smallest error ellipse [34]. The frequency of Argos location acquisition varied between years and tag types (e.g., collar vs ear tag). Argos acquisition varied between 1 and 4 days, with average durations between acquisition being 3 (SD = 3.1), 3 (SD = 1.4), and 2 days (SD = 1.0) for adult females, adult males, and sub-adults, respectively. Further, we removed individuals from the analysis that had < 3 locations, which is the minimum number of locations needed to estimate movement metrics. Unequal sampling among individuals was accommodated by our hierarchical modeling approach (see below). Lastly, we restricted our analyses to movement and step selection while on sea ice and not land as most bears are on sea ice in spring. Only denning bears would be on land for prolonged periods during spring and as stated earlier, we did not included bears with cubs-of-the-year in our analysis.

Due to computational constraints, we chose to use a data imputation approach to account for uncertainty in locations [35] rather than directly accounting for it in a state-space model (e.g., [34]). Following the guidance of Scharf et al. [35], for each individual’s location data we fit a continuous time correlated random walk model (CTCRW; [10]) that incorporated the location uncertainty estimated by Argos error ellipses [34] with the ‘crawl’ package [36] for R [37]. We then obtained 25 realizations for each animal’s estimated path, with estimated locations obtained at 4-day intervals, which we used for the subsequent analyses of movements and step selection. The CTCRW model was also able to account for missing Argos fixes for an individual, which would lead to greater variability in estimated locations further from observed locations. Twenty-five datasets were then created such that each dataset had one realized path from each individual (without replacement). We provide a general overview of the data imputation approach in Additional file 1.

### Movement analysis

We were interested in determining if there were differences in movement characteristics across different classes of polar bears to help inform movement models used to address management and conservation concerns [23, 38] and to gain a better understanding of the ecological differences among polar bears. Recently methods have been developed to jointly estimate movement metrics and step selection parameters jointly, termed integrated step selection analysis [39]. While our study objectives lend themselves to the use of this approach, we preferred to take a two-step approach (i.e., estimating movement and step selection parameters independently) for a variety of reasons. Given the limited sample sizes for some individuals in our study, especially adult males and sub-adults because of tag retention and performance issues [15], we wanted to take advantage of a trait of Bayesian hierarchical models that allows for borrowing strength across samples with varying levels of information [40]. This is not currently possible with the integrated step selection approach which would have likely required us to omit data from a number of individuals for our analysis. Additionally, integrated step selection analyses generally prefers separate models to be fit to each individual which are then averaged to develop population-level estimates [39]. Given our data imputation approach to handle location uncertainty, this would have led to 3,200 unique models needing to be run. We therefore developed a simple movement model to evaluate step length and directional persistence differences across the three classes studied and then conducted a separate step selection analysis (detailed below). We note, however, that by not taking an integrated step selection analysis approach, we are unable to correct the movement parameters to remove the influence of resource selection, which may lead to some bias in sample of available points [41].

Our use of a data imputation approach allowed us to obtain expected location data for all individuals at fixed time steps, which reduced model complexity compared to other state-space approaches [42–44]. Similarly, the data imputation approach eliminated the need to account for uncertainty associated with observed locations in the hierarchical model (see below), further simplifying the model.

We obtained 4-day step lengths and turn angles (i.e., distance traveled and turn angles between locations obtained at 4-day intervals) from the CTCRW output for each individual and used these values as data in our model. We modeled an individual’s step lengths as coming from a Weibull distribution:

$${l}_{i,t}\sim Weib\left({k}_{i},{g}_{i}\right)$$

where $l_{{i,t}}$ is the observed step length of individual *i* at time *t*, and ${k}_{i}$ and ${g}_{i}$ are individual-level shape and scale parameters, respectively, for individual *i* in the Weibull distribution. We used the method-of-moments approach [40] to parameterize the model to estimate individual-level parameters based on population-level parameters for each of the three classes:

$$\left[\left.{g}_{i}\right|{c}_{i}\right]\sim Gamma\left(\frac{{\gamma }_{c}^{2}}{{\sigma }_{\gamma }^{2}}, \frac{{\gamma }_{c}}{{\sigma }_{\gamma }^{2}}\right)$$

$$\left[\left.{k}_{i}\right|{c}_{i}\right]\sim Gamma\left(\frac{{\kappa }_{c}^{2}}{{\sigma }_{\kappa }^{2}}, \frac{{\kappa }_{c}}{{\sigma }_{\kappa }^{2}}\right)$$

where *c*_*i*_ is the class for individual *i* (i.e., adult female, adult male, sub-adult), ${\kappa }_{c}$ and ${\gamma }_{c}$ are population-level shape and scale parameters (specific for each class of bear), respectively, for the Weibull distribution of step lengths, and ${\sigma }_{\kappa }^{2}$ and ${\sigma }_{\gamma }^{2}$ represent variation among individuals for the two population-level parameters. We calculated the mean expected step length for class c as: ${\mu }_{c}={\gamma }_{c}\Gamma (1+1/{\kappa}_{c})$, where Γ is the log gamma function.

We modeled an individual’s turn angles as coming from a wrapped Cauchy distribution:

$${\theta }_{i,t}\sim wCauchy\left({\theta }_{i,t-1},{r}_{i}\right)$$

where ${\theta }_{i,t-1}$ is the turn angle for individual *i* at the previous time step (i.e., *t-1*) and ${r}_{i}$ is individual *i’s* estimated directional persistence. We again used the method-of-moments approach to estimate an individual’s directional persistence based on population-level parameters for each of the three classes:

$$\left[\left.{r}_{i}\right|{c}_{i}\right]\sim Beta\left(\frac{{\phi }_{c}^{2}-{\phi }_{c}^{3}-{\phi }_{c}{\sigma }_{\phi }^{2}}{{\sigma }_{\phi }^{2}},\frac{{\phi }_{c}-2{\phi }_{c}^{2}+{\phi }_{c}^{3}-{\sigma }_{\phi }^{2}+{\phi }_{c}{\sigma }_{\phi }^{2}}{{\sigma }_{\phi }^{2}}\right)$$

where ${\phi }_{c}$ is the population-level mean for directional persistence specific to each class of bear, and ${\sigma }_{\phi }^{2}$ is the inter-individual variation in directional persistence. We gave all population-level parameters vague priors, with ${\gamma }_{c}\sim Uniform\left(\mathrm{0,500000}\right)$, ${\kappa }_{c}\sim Uniform(\mathrm{0,10})$, ${\phi }_{c}\sim Uniform\left(\mathrm{0,1}\right)$, ${\sigma }_{\gamma }\sim Uniform(0, 50000)$, ${\sigma }_{\kappa }\sim Uniform (\mathrm{0,5})$, and ${\sigma }_{\phi }\sim Uniform(0, 0.5)$. Because the model for individual turn angles required the previous turn angle as a parameter, we also estimated the starting (i.e., *t* = 1) step length and turn angle as vague priors, with ${s}_{i,1}\sim Uniform\left(\mathrm{0,100000}\right)$ and ${\theta }_{i,1}\sim Uniform(-\pi ,\pi )$.

### Step selection analysis

To assess differences in polar bear step selection among classes, we developed step selection functions (SSFs) at the scale of 4-day step lengths. For each of the 25 imputed data sets, we estimated SSFs with a hierarchical Bayesian conditional logistic regression model [18, 45], which accounted for the lack of independence among multiple clusters (i.e., one used location with multiple random locations; see below) for individual bears. We obtained samples of available locations for each individual *i*’s used locations by first drawing a random sample from the individual’s posterior distributions for the shape (i.e., *k*_*i*_), scale (i.e., *g*_*i*_), and directional persistence (i.e., *r*_*i*_) parameters. We then obtained 25 random samples of step length from a Weibull distribution with the values of the sampled shape and scale parameters, and 25 random samples of turn angles from a Wrapped Cauchy distribution with the values of the previous turn angle for individual *i* (i.e., *θ*_*i,t-1*_) and the sample directional persistence parameter. We defined a used location with its associated available locations as a cluster. A sample of > 20 available locations has been shown to be sufficient for obtaining accurate estimates of habitat selection [46]. We generated available locations as:

$${{\varvec{v}}}_{{\varvec{t}},{\varvec{a}}}= {{\varvec{s}}}_{{\varvec{t}}-1}+\boldsymbol{ }\left(\genfrac{}{}{0pt}{}{{l}_{a}\mathrm{cos}{\theta }_{a}}{{l}_{a}\mathrm{sin}{\theta }_{a}}\right)$$

where ***v***_***t,a***_ is a two-element vector with an available location *a*’s x- and y-coordinates at time *t*, which is a function of the used location at the previous time step (${{\varvec{s}}}_{{\varvec{t}}-1}$; similarly a two-element vector with x- and y-coordinates) and a randomly sampled step length (${l}_{a}$) and turn angle (${\theta }_{a})$ from the posterior distribution as described above. We estimated the choice probability [47] for the used location, ${{\varvec{s}}}_{{\varvec{i}},{\varvec{t}}}$ for individual *i* at time *t* as:

$$\lceil{{\varvec{s}}}_{{\varvec{i}},{\varvec{t}}}|{{\varvec{s}}}_{{\varvec{i}},{\varvec{t}}-1},\boldsymbol{ }{{\varvec{s}}}_{{\varvec{i}},\boldsymbol{ }{\varvec{t}}-2}, {{\varvec{x}}}_{i,t}\rceil= \frac{{e}^{{\boldsymbol{\alpha }}_{{\varvec{i}}}{{\varvec{x}}}_{{\varvec{s}}}}}{\sum_{r\in {S}_{t}}{e}^{{\boldsymbol{\alpha }}_{{\varvec{i}}}{{\varvec{x}}}_{{\varvec{r}}}}}$$

where ${{\varvec{s}}}_{{\varvec{i}},{\varvec{t}}-1}$ is individual *i*’s used location at time *t-1*, ${{\varvec{x}}}_{i,t}$ is a vector of attributes corresponding to the set of used and available locations for individual *i* at time *t, S*_*t*_ is the set of all available and used points for individual *i* at time *t*, **x**_**s**_ is a vector of attributes corresponding to used location ${{\varvec{s}}}_{{\varvec{i}},{\varvec{t}}}$ (see below), and ***α***_***i***_ is a vector of parameters for animal *i* (i.e., individual-level selection parameters). We modeled each individual selection parameter as:

$${\alpha }_{i}\sim Normal({\beta }_{c},{\sigma }_{\beta })$$

where *β*_*c*_ represents the corresponding population-level selection parameter for class *c*, and ${\sigma }_{\beta }$ represents the inter-individual standard deviation for that selection parameter. To simplify the model, we assumed that ${\sigma }_{\beta }$ did not vary among classes. We assigned all *β*_*c*_ a vague prior, *β*_*c*_ ~ Normal(0,100). Similarly, we assigned all ${\sigma }_{\beta }$ a vague prior, ${\sigma }_{\beta }$ ~ Gamma(0.01,0.01).

We estimated selection using covariates that have previously been identified as important for describing polar bear resource selection patterns [18, 19]. Additionally, the following variables might be expected to have different relationships with other classes of polar bears given their connection to prey distribution [48] and observations that some classes may be more likely to occur near communities [25, 26] and therefore closer to land. Specifically, we estimated selection patterns across classes for ocean depth (resolution = 16 km^2^; http://dx.doi.org/10.7289/V5C8276M, accessed 17 March 2022); sea-ice concentration; sea-ice concentration-squared; the standard deviation of sea-ice concentration within 100 km of points, as an index of sea-ice variability [18]; distance to land; distance to land-squared; distance to the 10% ice concentration contour, as an index of the distance to the sea-ice edge and related to prey distribution [48]; and distance to the 10% ice concentration contour-squared. For all sea ice-related variable, we obtained data from Cavalieri et al. [49]. We also used the sea ice concentration maps to define distance to land as those datasets also classified the presence of land. Sea ice concentration-derived layers had a spatial resolution of 625 km^2^ and were available daily, so we related them the specific day and year a location was obtained.

We scaled each variable by subtracting their overall means from the observed values and then dividing by their overall standard deviations. To assess the predictive capacity of the model we used a cross-validation approach designed for case–control analyses [50]. We withheld 20% of clusters to use as a test set in for each of the 25 imputed data sets and used the remaining 80% of the clusters as the training data set. For each location in the withheld cluster, we calculated the choice probability based on the parameters estimated from the training dataset. We then ranked the calculated choice probabilities within each cluster from lowest to highest, extracted the rank for each used location, and calculated the number of used locations that were ranked in each of the 26 bins (with bins 1 and 26 having the lowest and highest calculated choice probabilities, respectively, within the cluster). Finally, we used Spearman’s rank correlation of the bin counts to assess predictive capacity of the model. A high correlation value is expected for good-fitting models because used locations should rank higher (on average) than available locations.

### Model implementation

For the two analyses (i.e., movement and step selection), we estimated the posterior distribution for each parameter with Monte Carlo Markov Chains using the package ‘rjags’ [51] to run the program JAGS [52] from R [37]. We ran the 25 imputed data sets in parallel, allowing 5,000 iterations for the adaptation phase and a burn in of 100,000 and 10,000 iterations for the movement and step selection analyses, respectively. We then obtained 100,000 and 5,000 iterations from each chain for the movement and step selection analyses (respectively) and thinned each by 100 and 5 (respectively), resulting in a total of 1,000 samples from the posterior distribution. We visually assessed each parameter for convergence. We then combined the 1,000 posterior samples from each imputed data set for each analysis, resulting in a posterior sample of 25,000 which we used to estimating summary statistics for model parameters. Given that our primary study objective was to assess if differences exist in parameter estimates between polar bear classes we assessed if the 95% Credible Intervals (CI) for a given class’s parameter estimate overlapped with the other class’s 95% CIs.

## Results

We obtained location data from 118 unique bears, representing 128 unique bear years of data (hereafter bears). Five bears provided data from 2 separate years, one bear provided data from 3 separate years, and one bear provided data from 4 separate years. All but one of the bears with multiple years of data were adult females, the other was an adult male. Of the 128 bears, 78 were adult females, 32 were adult males, and 20 were sub-adults of both sexes. Bears had an average of 28.5 (SD = 17.7) Argos locations that were used in the CTCRW analysis. From the CTCRW output, there was a total of 1978 4-day steps, with an average of 15.5 (SD = 5.5) 4-day steps per individual bear. Adult females had an average of ~ 5 additional 4-day steps per individual (adult female: mean = 17.7, SD = 4.9; adult male: mean = 12.2, SD = 4.7; sub-adult: mean = 12.1, SD = 4.8) due to the lower tag retention and poorer performance of ear and glue-on tags.

### Movement analysis

We observed differences in movement metrics between adult male and adult females, but not between sub-adults and adult bears of either sex (Table 1, Additional file 2). Plots of the posterior distribution of the movement parameters show overlap between all three bear classes for the Weibull shape parameter, but little overlap between adult males and adult females for the Weibull scale parameter, step length, and directional persistence (Additional file 2). Adult males exhibited larger step lengths and lower directional persistence than adult females with minimal overlap in their respective 95% CI (Table 1). These differences did not exist between adult males and sub-adult bears. Similarly, 95% CI of movement parameters overlapped between adult females and sub-adult bears (Table 1).

**Table 1:** Parameter estimates (median and 95% Credible Intervals [CI]) for the analysis comparing movement differences between adult female, adult male, and sub-adult polar bears in the Chukchi Sea subpopulation

Variable	Class					
	Adult female		Adult male		Sub-adult	
	Median	95% CI	Median	95% CI	Median	95% CI
*κ*	1.79	1.68–1.90	1.76	1.57–1.96	1.77	1.55–2.01
*γ*	67,288	62,947–71,962	79,025	71,078–87,080	69,079	59,966–78,667
Step length	59,864	56,012–64,014	70,396	63,361–77,512	61,532	53,486–70,000
*ρ*	0.248	0.200–0.296	0.159	0.066–0.243	0.212	0.096–0.325

The *κ* (i.e., shape) and *γ* (i.e., scale) parameters are for the Weibull distribution component of the model, whereas *ρ* is for the wrapped Cauchy distribution component of the model. The mean expected step length (m $$)$$ is derived from the Weibull distribution parameters (i.e., step length = *γ*Γ(1 + 1/$$\kappa$$), where Γ is the log gamma function)

### Step selection analysis

**Fig. 2:**
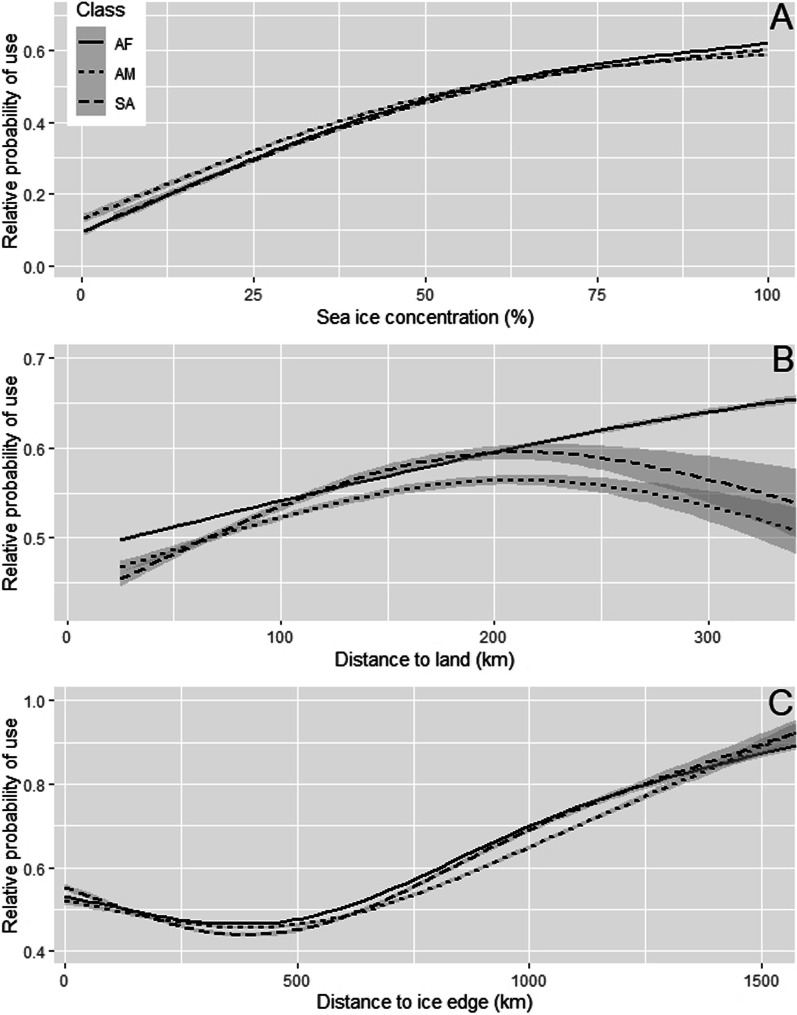
Average effects of sea ice concentration (**A**), distance to land (**B**), and distance to sea ice edge (**C**) on the relative probability of use for each of three classes (i.e., adult female [AF], adult male [AM], and sub-adult [SA]) of polar bears in the Chukchi Sea subpopulation in spring and early summer (i.e., 1 March–30 June). Plots are based on the habitat conditions present in the sample of available points used for the step selection analysis. We developed curves (and associated 95% Confidence Intervals of the smoothed curve; gray polygons) based on a smoothed (i.e., generalized additive model, with *df* = 3 for the sea ice concentration and distance to land variables, and *df* = 4 for the distance to ice edge variable) non-parametric model, as described in Avgar et al. [53]

Cross-validation of withheld data for our step selection model had a spearman rank correlation coefficient estimate of 0.95 (95% CI 0.90–0.98) indicating strong predictive capacity for the model. Selection coefficients across the three polar bear classes were similar, with only minor differences in estimates and largely overlapping credible intervals (Table 2, Additional file 2). Polar bears exhibited no selection for ocean depth but exhibited positive selection for more variable sea ice (Table 2), although sub-adults had 95% CI that slightly overlapped 0 (Table 2). Coefficient estimates for distance to land did not differ from zero for adult males and sub-adults, but was > 0 for adult females which showed selection for areas further from land (Fig. 2, Table 2, Additional file 2). All classes also showed decreasing selection for distance to the sea-ice edge until ~ 400 km, after which selection began to increase for further distances, but again with very wide credible intervals (Fig. 2, Table 2). Finally, all classes of polar bears showed increasing selection for more concentrated sea ice which stabilized around 80% concentration (Fig. 2, Table 2).

**Table 2:** Coefficient estimates (and associated 95% CI) for population-level parameters of a step selection model for three different classes of polar bears in the Chukchi Sea subpopulation

Variable	Class					
	Adult female		Adult male		Sub-adult	
	Median	95% CI	Median	95% CI	Median	95% CI
Depth	0.028	−0.065–0.142	0.031	−0.557–0.639	−0.046	−2.263–0.866
Conc	1.515	0.998–2.059	1.440	0.884–2.047	1.410	0.779–2.047
Conc^2^	−0.697	−1.260–0.166	−0.858	−1.545–0.262	−0.772	−1.446–−0.132
SDConc	0.236	0.095–0.377	0.308	0.099–0.522	0.223	−0.021–0.468
D2Land	0.485	0.005–0.960	0.488	−0.073–1.105	0.443	−0.165–1.055
D2Land^2^	−0.316	−0.832–0.206	−0.408	−1.099–0.206	−0.377	−1.105–0.318
D2Ice	−1.420	−2.356–0.469	−1.338	−2.327–−0.256	−1.367	−2.382–−0.236
D2Ice^2^	1.740	0.628–2.846	1.479	0.115–2.713	1.454	−0.153–2.730

Covariates included in the model were ocean depth (Depth), sea-ice concentration (Conc), ice concentration squared (Conc^2^), standard deviation in sea-ice concentration within a 100 km radius (SDConc), distance to land (D2Land), distance to land squared (D2Land^2^), distance to the pack ice edge (D2Ice) and distance to pack ice edge squared (D2Ice^2^). We considered variables whose 95% CI did not overlap 0 as statistically significant

## Discussion

Polar bears in the CS subpopulation exhibited different movement patterns across classes, which is largely consistent with findings from other subpopulations [20, 21]. Similar to Laidre et al. [21], we observed adult females having higher directional persistence in their movement compared to adult males. In their study, Laidre et al. [21] attributed this pattern to adult male movement being motivated by searching for potential mates in the spring, with less directed movements leading to lower encounter rates with competing males. This explanation is consistent with our results which were similarly based on movement data collected during the breeding season for polar bears [54]. Although sub-adult polar bear directional persistence did not differ from either adult bear class, their mean persistence was closer to adult female levels, likely indicating adult female and sub-adult bear movements are not influenced by breeding behavior. This is further supported by Laidre et al.’s [21] finding that reproductive status of a female had no relationship with directional persistence. Adult males had longer step length than other classes which is counter to our predictions and inconsistent with previous studies of polar bears [21].

We did not find meaningful differences in step selection patterns across demographic classes of bears in the CS subpopulation, which is consistent with other studies [21], despite the ecological differences between our study systems [55]. Variables in our analysis were primarily related to factors associated with accessibility to prey in relation to ice dynamics but did not include data on the distribution of prey due to a lack of availability. If these data become available they could potentially influence the results and highlight differences in selection given that males tend to prey more on bearded seals whereas adult females and younger bears predominantly prey on ringed seals [27, 56], both which have different distributions [28]. We predicted that adult males might display differences in step selection patterns from other classes of bears in our study given their greater reliance on bearded seal as prey [27]. The lack of difference between classes in our study might suggest that during spring, adult males are also capitalizing on ringed seal pups which are only available for a short period [57]. Conversely, adult males might be most focused on mating in the spring and thus matching their space use patterns to adult females. In either case, additional research is warranted during other periods of the year.

Our selection estimates for distance to ice edge (Fig. 2) reflect results from Von Duyke et al. [58] that ringed seal distribution in the CS region is negatively related to distance from ice edge, and from the results of Cameron et al. [48] who showed juvenile bearded seals selecting for areas closer to the ice edge. Our results indicated that bears showed decreased selection for areas further from the ice edge, out to ~ 400 km (Fig. 2). Although we also observed an increase in selection for distances beyond 400 km from the ice edge, this relationship was highly uncertain (Fig. 2) because the majority (58%) of locations were from bears < 550 km from the ice edge.

All classes of polar bears studied exhibited similar patterns of selection for areas further from land (Fig. 2). These results indicate that during late spring no class of polar bear is selecting for areas nearer to human settlements than other classes. Spring is the period of the year when ice is at its greatest extent and hunting success is highest [57], thus polar bears should be motivated to remain on the sea ice. The only activity in spring that would be likely to cause polar bears to occur near communities would be spring whaling activities which can provide food resources to polar bears. Our results indicate that the majority of bears in the CS subpopulation prefer to remain on the sea ice in spring. This does not mean that there are not differences in the level of conflict between humans and different classes of polar bears, as has been previously documented [25, 26]. Rather, human-polar bear conflict is most likely related to behavioral differences among polar bears [59].

Our analysis of movement and step selection at the scale of 4-day steps was primarily motivated by the resolution of spatial data from adult males and sub-adults and to compare with Laidre et al. [21] which also used 4-day steps for their analysis. We note, however, that the patterns we observed between classes could easily change with finer-scale movement data (if that ever becomes available for classes other than adult females). At a 4-day time scale, the three classes of bears we studied appear to be making similar decisions on where to move next. But this could mask differences in movement decisions at finer temporal scales. For example, if different classes of bears hunt in similar areas, but display differences in hunting strategies (e.g., breaking in ice seal lairs vs. ambushing hauled-out seals), these differences might not be detected with a 4-day step length. Similarly, it is well-known that estimated distances moved between two points in time becomes increasingly biased low with longer durations between those points [60]. With finer-scale movement data, we might have observed even larger relative differences in step lengths between classes or shown differences in directional bias based on different movement decisions being made during small time intervals. Thus, inference from our study can only be applied to questions that generally match the temporal scale of our movement data.

Our findings generally support the use of adult female step selection patterns during spring to inform the movement decisions of other classes of polar bears (e.g., [23]). Future research is warranted, however, when information becomes available on the abundance and distribution of polar bear prey species in the region. While spring is an important time for polar bears from a mating [54] and energetic perspective [57], our results might not be representative of other periods of the year. For example, adult male movement and selection patterns are likely tightly linked to adult female patterns given that our study period overlaps with polar bear breeding [54]. We know that during other periods of the year, adult female movements and space use decisions can be affected by their reproductive status, leading to differences compared to other classes of bears [22]. Additional research during other periods of the year is warranted if adult female movement and resource selection patterns are to be used as a proxy for other classes of bears that cannot be fitted with collars. The limited battery life of non-collar tags, however, would require captures to occur during other periods of the year (e.g., autumn) which can be challenging given differences in the distribution of bears (e.g., some portion the sub-population on land and some on ice) and difficulty in capturing bears adjacent to open water.

## Conclusions

We found some support for the use of adult female movement data to infer the spatial ecology of other classes of bears in the CS subpopulation. Careful consideration is required to determine whether such inference is warranted based on the questions being asked and the time period of interest. Our results highlight the need to evaluate class-specific movement and step selection during other periods of the year than spring. Results from our movement analysis show that relying on data from adult females as proxy for the movements of adult males is likely inappropriate, as has been documented previously for other populations [21]. This could be an especially important consideration in studies relying on simulated polar bear movements to estimate potential impacts of anthropogenic activities (e.g., oil spills [61]) or that assess the relative outcomes of different management or conservation activities on bears [38].

## Supplementary Information

**Additional file 1.** Description of multiple imputation approach for movement and step selection analyses.

**Additional file 2.** Plots of the posterior distribution of parameters estimated for the estimation of movement metrics and step selection coefficients.

## Data Availability

The datasets supporting the conclusions of this article are available in the Dryad repository, [https://doi.org/10.5061/dryad.g1jwstqs9].

## Abbreviations

CI: Credible interval
CS: Chukchi sea
CTCRW: Continuous time correlated random walk
GPS: Global positioning system
SSF: Step selection function

## References

[1] Merrick MJ, Koprowski JL (2017). Should we consider individual behavior differences in applied wildlife conservation studies?. Biol Conserv.

[2] Bacheler NM, Paramore LM, Burdick SM, Buckel JA, Hightower JE (2009). Variation in movement patterns of red drum (*Sciaenops ocellatus*) inferred from conventional tagging and ultrasonic telemetry. Fish Bull.

[3] Blanchard BM, Knight RR (1991). Movements of yellowstone grizzly bears. Biol Conserv.

[4] Rosenblatt AE, Heithaus MR, Mazzotti FJ, Cherkiss M, Jeffery BM (2013). Intra-population variation in activity ranges, diel patterns, movement rates, and habitat use of American alligators in a subtropical estuary. Estuar Coast Shelf Sci.

[5] Ajemian MJ, Drymon JM, Hammerschlag N, Wells RJD, Street G, Falterman B (2020). Movement patterns and habitat use of tiger sharks (*Galeocerdo cuvier*) across ontogeny in the Gulf of Mexico. PLoS ONE.

[6] Sandell M (1986). Movement patterns of male stoats *Mustela erminea* during the mating season: differences in relation to social status. Oikos.

[7] Allen RM, Metaxas A, Snelgrove PVR (2018). Applying movement ecology to marine animals with complex life cycles. Ann Rev Mar Sci.

[8] Olson MH (1996). Ontogenetic niche shifts in largemouth bass: variability and consequences for first- year growth. Ecology.

[9] Howell LN, Reich KJ, Shaver DJ, Landry AM, Gorga CC (2016). Ontogenetic shifts in diet and habitat of juvenile green sea turtles in the northwestern Gulf of Mexico. Mar Ecol Prog Ser.

[10] Johnson DS, London JM, Lea M-A, Durban JW (2008). Continuous-time correlated random walk model for animal temetry data. Ecology.

[11] Wilson RR, Horne JS, Rode KD, Regehr EV, Durner GM (2014). Identifying polar bear resource selection patterns to inform offshore development in a dynamic and changing Arctic. Ecosphere.

[12] Pinard V, Dussault C, Ouellet JP, Fortin D, Courtois R (2012). Calving rate, calf survival rate, and habitat selection of forest-dwelling caribou in a highly managed landscape. J Wildl Manage.

[13] Marchand P, Freycon P, Herbaux JP, Game Y, Toïgo C, Gilot-Fromont E (2017). Sociospatial structure explains marked variation in brucellosis seroprevalence in an Alpine ibex population. Sci Rep.

[14] Swan GJF, Redpath SM, Bearhop S, McDonald RA (2017). Ecology of problem individuals and the efficacy of selective wildlife management. Trends Ecol Evol.

[15] Wiig Ø, Born EW, Laidre KL, Dietz R, Jensen MV, Durner GM (2017). Performance and retention of lightweight satellite radio tags applied to the ears of polar bears (*Ursus maritimus*). Anim Biotelemetry.

[16] Atwood TC, Peacock E, McKinney MA, Lillie K, Wilson R, Douglas DC (2016). Rapid environmental change drives increased land use by an Arctic marine predator. PLoS ONE.

[17] Rode KD, Wilson RR, Regehr EV, Martin MS, Douglas DC, Olson J (2015). Increased land use by Chukchi sea polar bears in relation to changing sea ice conditions. PLoS ONE.

[18] Wilson RR, Regehr EV, Rode KD, St MM (2016). Invariant polar bear habitat selection during a period of sea ice loss. Proc R Soc B Biol Sci.

[19] Durner GM, Douglas DC, Nielson RM, Amstrup SC, Mcdonald TL, Stirling I (2009). Predicting 21st-century polar bear habitat distribution from global climate models. Ecol Monogr.

[20] Amstrup SC, Durner GM, McDonald TL, Mulcahy DM, Garner GW (2001). Comparing movement patterns of satellite-tagged male and female polar bears. Can J Zool.

[21] Laidre KL, Born EW, Gurarie E, Wiig Ø, Dietz R, Stern H (2013). Females roam while males patrol: Divergence in breeding season movements of pack-ice polar bears (*Ursus maritimus*). Proc R Soc B Biol Sci.

[22] Johnson AC, Derocher AE (2020). Variation in habitat use of Beaufort Sea polar bears. Polar Biol.

[23] Regehr EV, Hostetter NJ, Wilson RR, Rode KD, Martin MS, Converse SJ (2018). Integrated population modeling provides the first empirical estimates of vital rates and abundance for polar bears in the Chukchi Sea. Sci Rep.

[24] Durner GM, Douglas DC, Atwood TC (2019). Are polar bear habitat resource selection functions developed from 1985–1995 data still useful?. Ecol Evol.

[25] Towns L, Derocher AE, Stirling I, Lunn NJ, Hedman D (2009). Spatial and temporal patterns of problem polar bears in Churchill. Manitoba Polar Biol.

[26] Wilder JM, Vongraven D, Atwood T, Hansen B, Jessen A, Kochnev A (2017). Polar bear attacks on humans: implications of a changing climate. Wildl Soc Bull.

[27] Rode KD, Regehr EV, Douglas DC, Durner G, Derocher AE, Thiemann GW (2014). Variation in the response of an Arctic top predator experiencing habitat loss: feeding and reproductive ecology of two polar bear populations. Glob Chang Biol.

[28] Bengtson JL, Hiruki-Raring LM, Simpkins MA, Boveng PL (2005). Ringed and bearded seal densities in the eastern Chukchi Sea, 1999–2000. Polar Biol.

[29] Durner GM, Laidre KL, York GS. Proceedings of the 18th Working Meeting of the IUCN/SSC Polar Bear Specialist Group. Gland, Switzerland: IUCN; 2018. p. 207.

[30] Stern HL, Laidre KL (2016). Sea-ice indicators of polar bear habitat. Cryosphere.

[31] Stirling I, Spencer C, Andriashek D (1989). Immobilization of polar bears (Ursus maritimus) with Telazol in the Canadian Arctic. J Wildl Dis.

[32] Rode KD, Pagano AM, Bromaghin JF, Atwood TC, Durner GM, Simac KS (2014). Effects of capturing and collaring on polar bears: findings from long-term research on the southern Beaufort Sea population. Wildl Res.

[33] Irvine LM, Winsor MH, Follett TM, Mate BR, Palacios DM (2020). An at-sea assessment of Argos location accuracy for three species of large whales, and the effect of deep-diving behavior on location error. Anim Biotelemetry.

[34] Mcclintock BT, London JM, Cameron MF, Boveng PL (2015). Modelling animal movement using the Argos satellite telemetry location error ellipse. Methods Ecol Evol.

[35] Scharf H, Hooten MB, Johnson DS (2017). Imputation approaches for animal movement modeling. J Agric Biol Environ Stat.

[36] Johnson DS, London JM. crawl: an R package for fitting continuous-time correlated random walk models to animal movement data. 2018.

[37] R Core Team. R: a language and environment for statistical computing. Vienna, Austria: R Foundation for Statistical Computing; 2021.

[38] Crevier LP, Salkeld JH, Marley J, Parrott L (2021). Making the best possible choice: Using agent-based modelling to inform wildlife management in small communities. Ecol Modell.

[39] Avgar T, Potts JR, Lewis MA, Boyce MS (2016). Integrated step selection analysis: Bridging the gap between resource selection and animal movement. Methods Ecol Evol.

[40] Hobbs NT, Hooten MB (2015). Bayesian models: A statistical primer for ecologists. Bayesian Model A Stat Prim Ecol.

[41] Street GM, Rodgers AR, Avgar T, Vander Vennen LM, Fryxell JM (2017). Comparing resource selection and demographic models for predicting animal density. J Wildl Manage.

[42] Mcclintock BT, Russell DJF, Matthiopoulos J, King R (2013). Combining individual animal movement and ancillary biotelemetry data to investigate population-level activity budgets. Ecology.

[43] Morales JM, Haydon DT, Frair J, Holsinger KE, Fryxell JM (2004). Extracting more out of relocation data: building movement models as mixtures of random walks. Ecology.

[44] Jonsen ID, Flemming JM, Myers RA (2005). Robust state-space modeling of animal movement data. Ecology.

[45] Thomas DL, Johnson D, Griffith B, Geological US (2006). A Bayesian random effects discrete-choice model for resource selection: population-level selection inference. J Wildl Manage.

[46] Northrup JM, Hooten MB, Anderson CR, Wittemyer G (2013). Practical guidance on characterizing availability in resource selection functions under a use-availability design. Ecology.

[47] Lele SR, Merrill EH, Keim J, Boyce MS (2013). Selection, use, choice and occupancy: clarifying concepts in resource selection studies. J Anim Ecol.

[48] Cameron MF, Frost KJ, Ver Hoef JM, Breed GA, Whiting AV, Goodwin J (2018). Habitat selection and seasonal movements of young bearded seals (*Erignathus barbatus*) in the Bering Sea. PLoS ONE.

[49] Cavalieri DJ, Parkinson CL, Gloersen P, Zwally HJ. Sea ice concentrations from Nimbus-7 SMMR and DMSP SSM/I-SSMIS passive microwave data, Version 1. Boulder, CO, USA; 1996.

[50] Daniel F, Fortin ME, Beyer HL, Thierry D, Sabrina C, Dancose K (2009). Group-size-mediated habitat selection and group fusion-fission dynamics of bison under predation risk. Ecology.

[51] Plummer M. rjags: Bayesian graphical models using MCMC. 2019.

[52] Plummer M. JAGS: a program for analysis of Bayesian graphical models using Gibbs sampling. In: Hornik K, Leisch F, Zeileis A, editors. Proceedings of the 3rd International Workshop on Distributed Statistical Computing. Technische Universitaet Wien, Vienna, Austria; 2003.

[53] Avgar T, Lele SR, Keim JL, Boyce MS (2017). Relative selection strength: quantifying effect size in habitat- and step-selection inference. Ecol Evol.

[54] Amstrup SC, Feldhamer GA, Thompson BC, Chapman JA (2003). Polar Bear. Wild Mammals of North America: biology, management, and conservation.

[55] Amstrup SC, Marcot BG, Douglas DC (2008). A bayesian network modeling approach to forecasting the 21st century worldwide status of polar bears. Geophys Monogr Ser.

[56] Rode KD, Regehr EV, Bromaghin JF, Wilson RR, St M, Justin M (2021). Seal body condition and atmospheric circulation patterns influence polar bear body condition, recruitment, and feeding ecology in the Chukchi Sea. Global Change Biol.

[57] Rode KD, Wilson RR, Douglas DC, Muhlenbruch V, Atwood TC, Regehr EV (2018). Spring fasting behavior in a marine apex predator provides an index of ecosystem productivity. Glob Chang Biol.

[58] Von Duyke AL, Douglas DC, Herreman JK, Crawford JA (2020). Ringed seal (*Pusa hispida*) seasonal movements, diving, and haul-out behavior in the Beaufort, Chukchi, and Bering Seas (2011–2017). Ecol Evol.

[59] Atwood TC, Wilder JM, Davis RW, Pagano AM (2021). Human-polar bear interactions. Ethology and behavioral ecology of sea otters and polar bears.

[60] Prichard AK, Yokel DA, Rea CL, Person BT, Parrett LS (2014). The effect of frequency of telemetry locations on movement-rate calculations in arctic caribou. Wildl Soc Bull.

[61] Wilson RR, Perham C, French-McCay DP, Balouskus R (2018). Potential impacts of offshore oil spills on polar bears in the Chukchi Sea. Environ Pollut..

