# Supplementary material for: Intrapopulation differences in polar bear movement and step selection patterns
Source: Mov Ecol. 2022 May 23;10:25. doi: 10.1186/s40462-022-00326-5 (PMC9128121; doi:10.1186/s40462-022-00326-5)
Supplement: Supplementary file 1 — Additional file 1. Description of multiple imputation approach for movement and step selection analyses. [file 40462_2022_326_MOESM1_ESM.pdf]

Additional file 1. Description of multiple imputation approach for movement and step selection analyses.

Implementing the multiple imputation approach outlined by Scharf et al. (1, 2) is a straightforward technique to propagate location uncertainty into model results while not having to explicitly model it in a state-space framework (which can be computationally demanding). Below we list the steps we took in this study to obtain the imputations and how we integrated them into the models for estimating movement and step selection parameters.

1. Format movement data (and associated location error data) according to specifications for use in the *cwrMLE* function in the 'crawl' package (3) for R (4).
2. For each animal's movement data, fit the continuous-time correlated random walk model using the *crwMLE* function.
3. Choose the time interval you wish to predict locations at and then use the fitted *crwMLE* object to construct a posterior simulation object in 'crawl' using the *crwSimulator* function.
4. Using the posterior simulation object, obtain *n* random draws from the posterior path distribution with the *crwPostIS* function.
5. Extract the predicted locations from each of the *n* posterior track samples.
6. Use these *n* posterior draws to calculate movement metrics (e.g., turn angles, step lengths) or as samples of used locations for step selection analyses.
7. Run separate MCMC algorithms for each of the *n* sampled tracks
8. Combine posterior samples from the *n* MCMC runs and draw inference from the combined posterior samples.

We refer readers to Scharf et al. (1, 2) for additional details on the implementation of this method for dealing with measurement error in movement data.

Literature Cited

(1) Scharf H, Hooten MB, Johnson DS. Imputation approaches for animal movement modeling. J Agric Biol Environ Stat. 2017;22:335–52.
(2) Scharf H, Hooten MB, Wilson RR, Durner GM, Atwood TC. Accounting for phenology in the analysis of animal movement. Biometrics 2019;75:810–820.
(3) Johnson DS, London JM. crawl: an R package for fitting continuous-time correlated random walk models to animal movement data. 2018.
(4) R Core Team. R: a language and environment for statistical computing. Vienna, Austria: R Foundation for Statistical Computing; 2021.
