# Supplementary material for: Intrapopulation differences in polar bear movement and step selection patterns
Source: Mov Ecol. 2022 May 23;10:25. doi: 10.1186/s40462-022-00326-5 (PMC9128121; doi:10.1186/s40462-022-00326-5)

Additional file 2. Plots of the posterior distribution of parameters estimated for the estimation of movement metrics (i.e., Weibull shape and scale parameters, step length, directional persistence) and step selection coefficients. Although the total number of posterior samples used in the study was 25,000, we subsampled the posterior distributions to obtain 1,000 samples to better show differences in parameter estimates between polar bear classes. We also provide plots of the posterior densities for each parameter, based on the entire posterior sample (i.e., 25,000 samples).


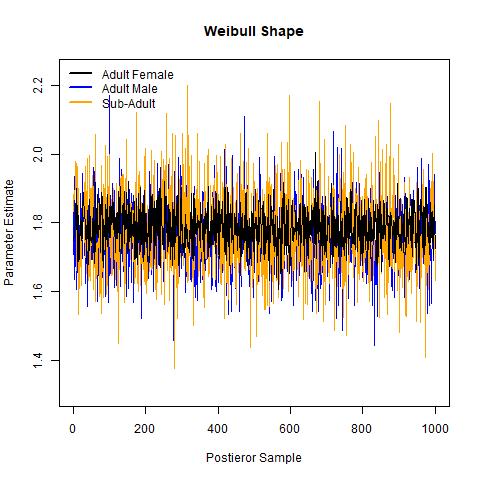
*Movement parameters*


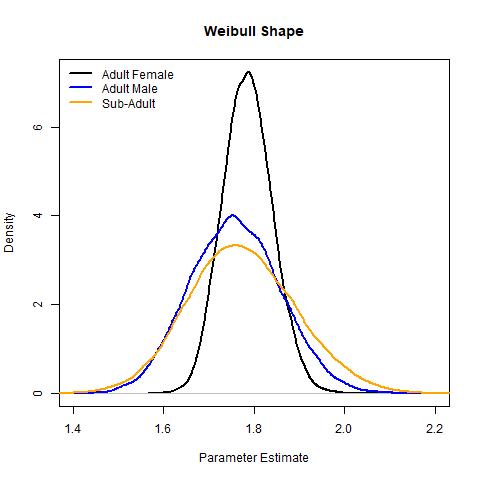


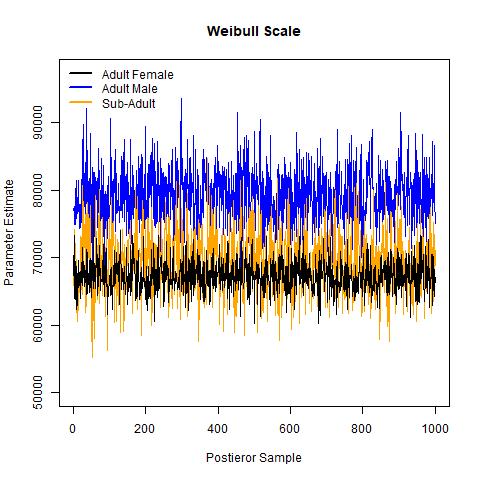


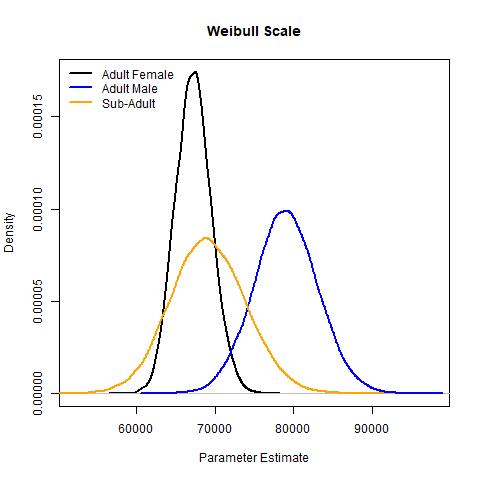


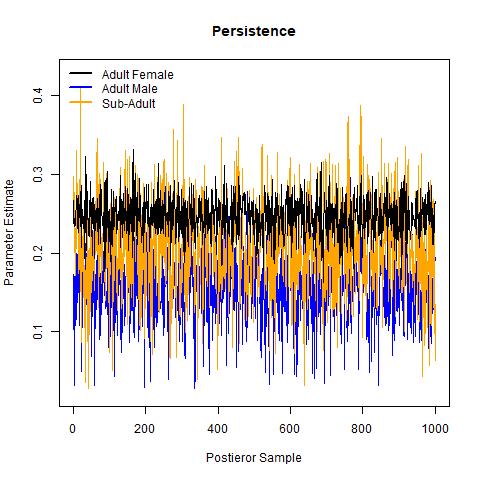


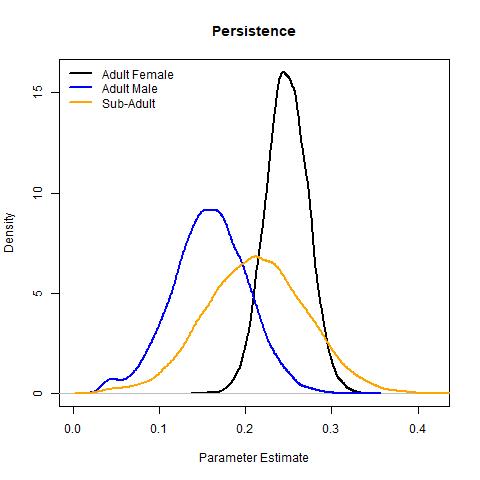


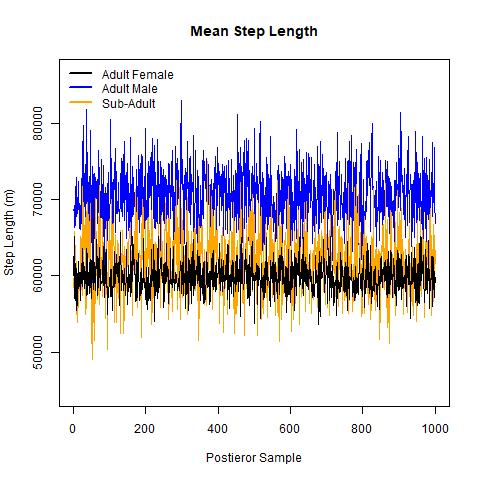


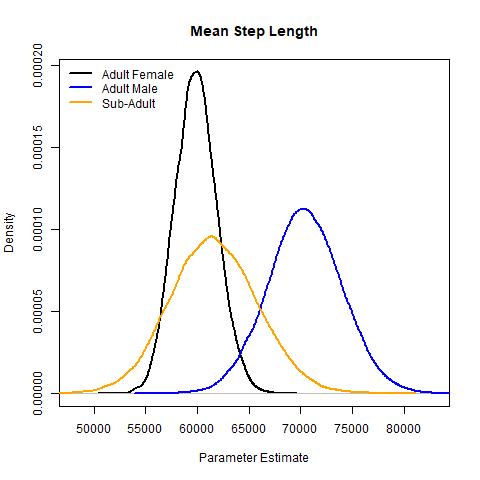


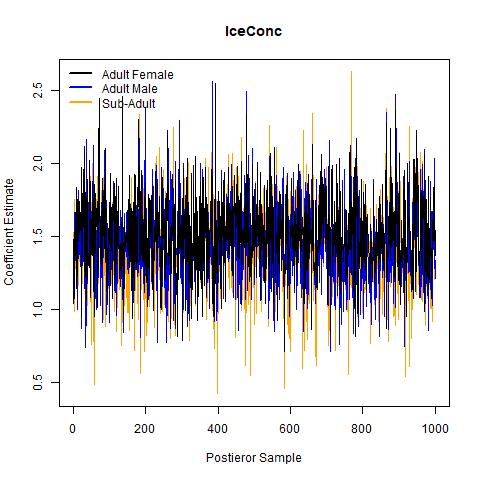
*Step selection parameters*


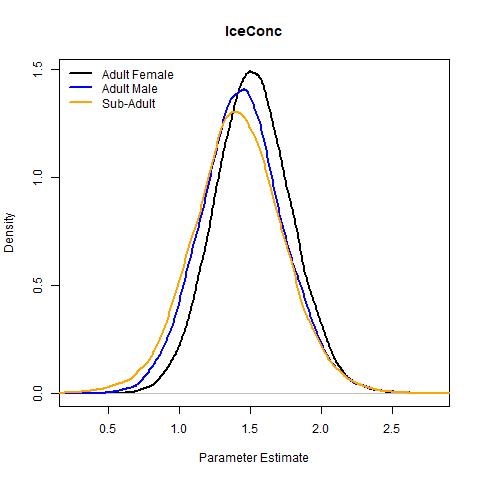


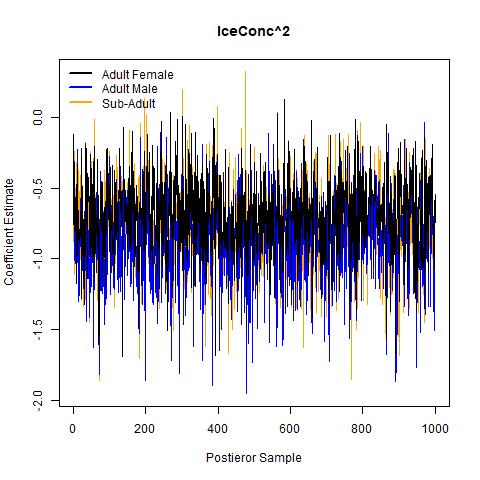


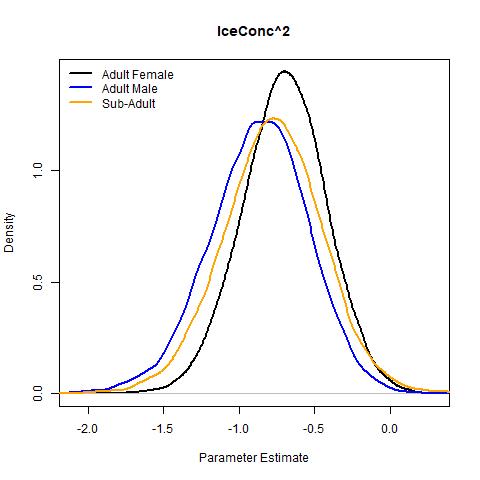


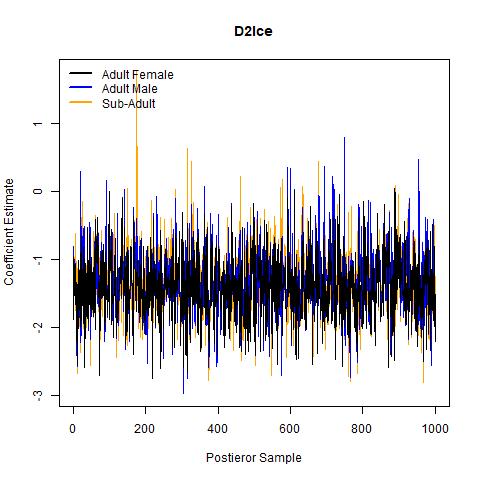


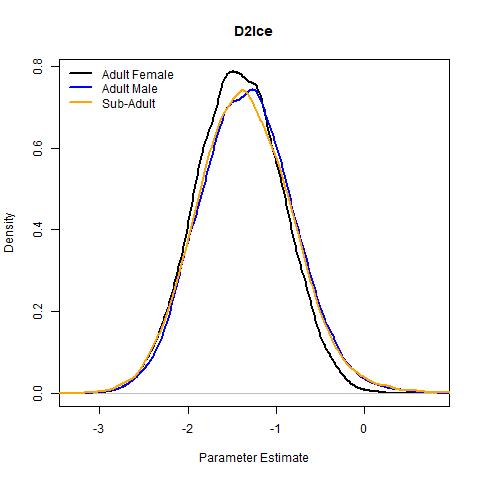


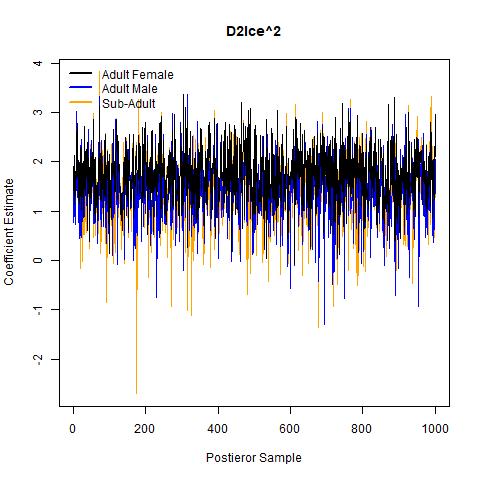


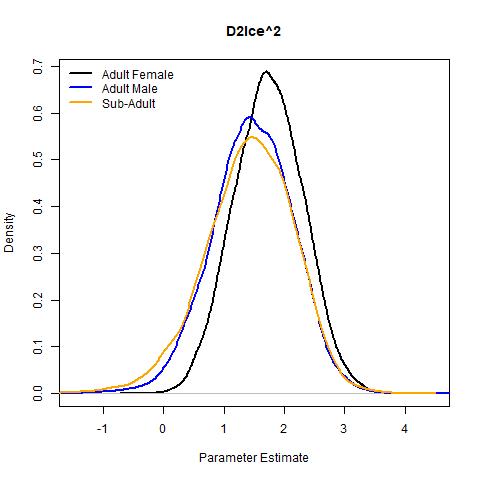


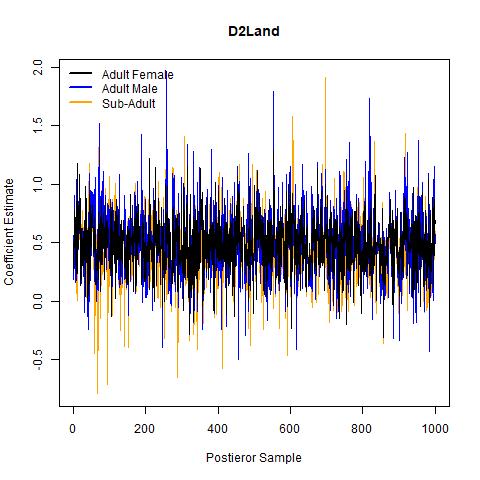


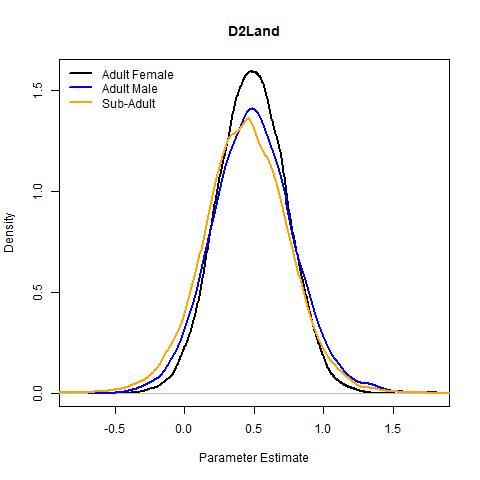


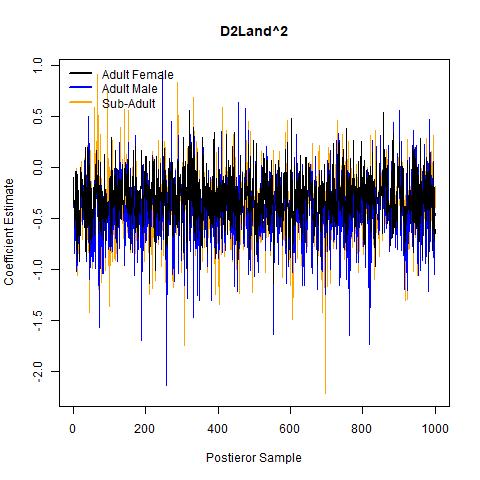


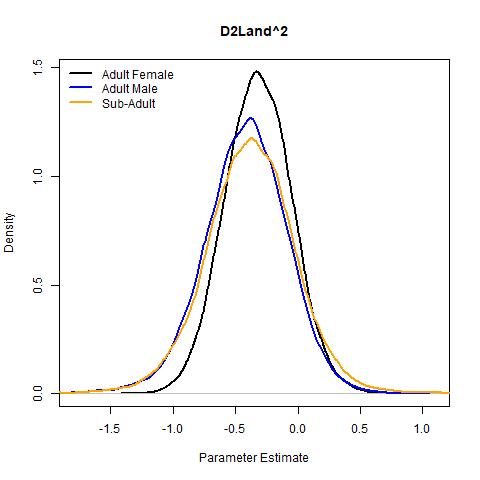


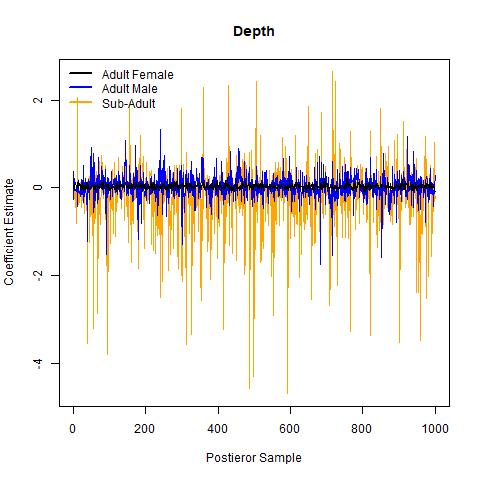


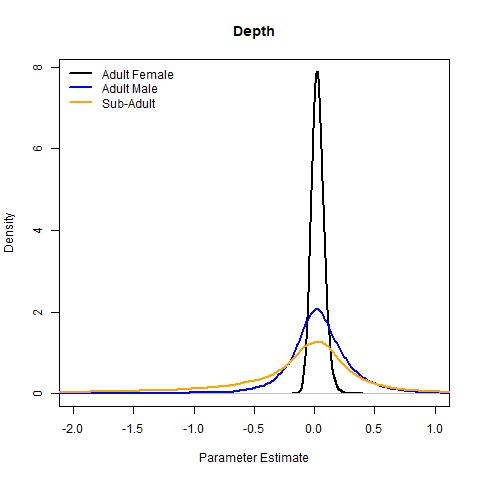


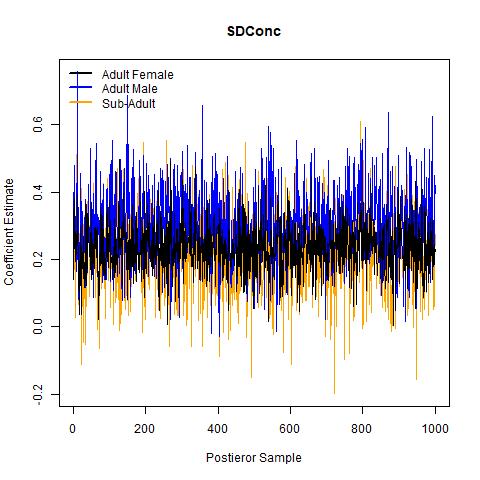


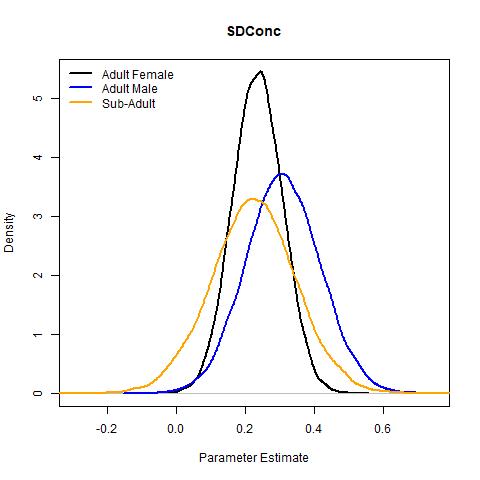

Supplement: Supplementary file 2 — Additional file 2. Plots of the posterior distribution of parameters estimated for the estimation of movement metrics and step selection coefficients. [file 40462_2022_326_MOESM2_ESM.docx]
